# Comparison of CO breath testing and women's self-reporting of smoking behaviour for identifying smoking during pregnancy

**DOI:** 10.1186/1747-597X-3-4

**Published:** 2008-02-17

**Authors:** Zara C Usmani, Pauline Craig, Deborah Shipton, David Tappin

**Affiliations:** 1 PEACH Unit, Department of Child Health, University of Glasgow, 8th Floor Tower, Queen Mother Hospital, Yorkhill, Glasgow, UK; 2 Glasgow Centre for Population Health, 39 St Vincent Place, Glasgow, UK; 3 Social & Public Health Sciences Unit, Medical Research Council, Glasgow, UK

## Abstract

**Background:**

Healthcare services often use a carbon monoxide (CO) breath test to validate self-reported smoking and to assess reductions in smoking habit. A cut-off level of ≥ 8 parts per million (p.p.m.) is used to identify smoking. This cut-off requires further validation in pregnant women.

**Methods:**

Data on self-reported smoking were assessed in conjunction with breath CO levels. Subjects in the study were 2548 women attending antenatal booking during 12 months.

**Results:**

546/2584 (21.4%) women self-reported as current smokers. A cut-off of 8 ppm identified only 325/546 self-reported smokers (sensitivity 59.4%). 27/2002 self-reported non-smokers had levels greater than 8 ppm (specificity 98.7%). Sensitivity and specificity analysis revealed that CO cut-off levels of 2 or 3 p.p.m. resulted in the best sensitivity and specificity for discriminating apparent smokers and non-smokers. A cut-off of 2 p.p.m. would have identified 468/546 of self-reported smokers (sensitivity 86%). 206/2002 self-reported non-smokers had levels > 2 ppm (specificity 90 %). If all these women were 'true' smokers, the real prevalence of smoking in pregnancy was 26.5% (752/2548) and 27% of true smokers provided false answers to the self-reported question at maternity booking.

**Conclusion:**

At 8 ppm, many smokers are missed and there may be gross underestimating of levels of smoking in a pregnant population. Results emphasise the need to support a lower cut-off level for the breath CO test closer to 2 or 3 p.p.m. These cut-offs may be more appropriate in the antenatal clinic setting, and are in line with recent recommendations in the non-pregnant population.

## Background

Many studies have attempted to assess the effects of smoking during pregnancy on maternal and child complications. Adverse consequences, including premature birth, low birth weight and long-term sequelae including developmental problems such as cognitive delays have been connected to maternal smoking [1,2].

A variety of factors have contributed to making it difficult to evaluate the effects of smoking during pregnancy. These include errors in measurement such as maternal denial and under-reporting, fluctuating behaviour of smoking during pregnancy and metabolism, including accelerated metabolism during pregnancy [3].

It is important clinically to have knowledge of patients smoking habits since it enables appropriate anti-smoking advice to be given and pregnancy is seen as a window of opportunity to provide this. Meta-analyses, including a Cochrane review, show that appropriate screening plus active intervention, in the form of advice and provision of written materials, increases the numbers of pregnant women who stop smoking. This in turn can reverse the adverse effects of smoking on perinatal outcomes by up to 20% [4,5]. It has also been emphasised that, in pregnancy, biochemical validation can increase women's motivation to stop smoking and increase their utilization of available treatment services [6], though a recent review showed no evidence that biofeedback motivated cessation [7].

In the general population, the proportion of people who report to be non-smokers but show biochemical levels indicative of smoking are generally low [8]. In pregnancy, however, women who smoke can experience intense social and clinical pressure that results in false declarations of non-smoking. This inaccuracy of self-reported smoking makes appropriate counselling difficult and stresses the need for reliable biochemical confirmation of smoking status [9].

Of the biochemical measures to assess smoking, cotinine, a metabolite of nicotine found in the blood and urine, is most preferred by scientists because of its long half-life, averaging 17 hr in non-pregnant women [10] and 9 hr in pregnant women [11]. In the clinical setting however, breath carbon monoxide (CO) level is seen to provide an immediate, non-invasive method of assessing smoking status and is the method most suitable to the antenatal clinic [12]. It is less preferred as a biochemical marker because CO has a short half-life of approximately 1–4 hours [13] and may not detect low levels of smoking [14]. Furthermore, the development of relatively inexpensive portable CO monitors enables breath CO levels to be assessed in a wide variety of clinical settings [15].

Currently in Glasgow, all women attending the antenatal clinic are CO monitored and those with a reading of ≥ 8 ppm or self-reported smokers are directly referred to a smoking cessation link midwife for a 6 week support programme. Since the results of the CO test and or self-report are the critical factors for referral for cessation advice, the accuracy of these is paramount to providing intervention. The 8 ppm cut-off in this programme is based on a standard cut-off for abstinence verification that is widely accepted within the research community [16]. New evidence suggests that this may not be appropriate in all settings. Recently, Javors et al concluded that the cut-off should be lowered to 2 or 3 ppm for assessment of smoking in a general population and a cut-off of 4 ppm in post partum women was suggested [17,18].

The present study examines the sensitivity and specificity of the breath CO test, and the optimal cut-off level to distinguish smokers from non-smokers amongst pregnant women.

## Methods

Data was collected at the antenatal booking appointment when women have a form completed by a nursing auxiliary along with the results of a CO breath test. These forms were obtained from the office for the Scottish smoking intervention programme called 'Breathe', in the Southern General Maternity Unit, Glasgow. Ethical approval was obtained from the Local Research Ethics Committee.

The Southern General Hospital has an 85–90% rate of return for the Breathe service forms of all women booked; the results of the present report are therefore likely to be applicable to the general pregnant population. Women who provided relevant Breathe forms and booked for antenatal care in the Southern General Hospital Glasgow between the months of July 2005 and June 2006 were included in this study.

The cut-off level for a CO test indicates that a result at or above (≥) the cut-off level would be a positive test for a smoking day (presumed to have smoked). A CO level below the cut-off would be a negative test (presumed a non smoker). In this study, various CO cut-off levels were used to evaluate cut-off levels for their accuracy in identifying pregnant smokers and non smokers. For example, CO level of 3 p.p.m. would be a positive test at a CO cut-off of 3 p.p.m.

### Study Procedure

All Breathe forms for women who booked between the months of July 2005 and June 2006 were inspected. Data on these forms is recorded by a midwife or auxiliary nurse at the antenatal booking clinic. At the clinic, the CO test is offered at a point suited to the midwife/auxiliary nurse and the test itself is performed using the EC50 Smokerlyzer (Bedfont Instruments; Kent, UK), an inexpensive, portable CO monitor that has previously been shown to be effective [15]. Midwives and auxiliary nurses have a two hour accredited training session regarding use of the device.

Data extracted from the forms included: self-reported smoking status, CO levels measured at the booking visit, date of birth, date of booking, number of cigarettes smoked per day and any additional comments made by the women.

### Statistical methods

Self-reported smoking and CO validated smoking status were analysed descriptively, using sensitivity and specificity percentages. Sensitivity was defined as the percentage of positive CO tests (a CO level at or above a given cut-off) for self-reported smokers, i.e. the percentage of all self-reported smokers for which there was a positive CO test. Specificity was defined as the percentage of negative CO tests (a CO level below a given cut-off) for self-reported non-smokers, i.e. percentage of all non-smokers for which there was a negative CO test. (Sensitivity + specificity) divided by 2 was calculated for each possible cut-off to identify the CO cut-off level that would have the highest average of combined sensitivity plus specificity.

Data were analysed by the SPSS 14 and Minitab 14 statistical packages.

## Results

### Levels of smoking, and sensitivity, specificity of self-report against carbon monoxide (CO)

**Figure 1:**
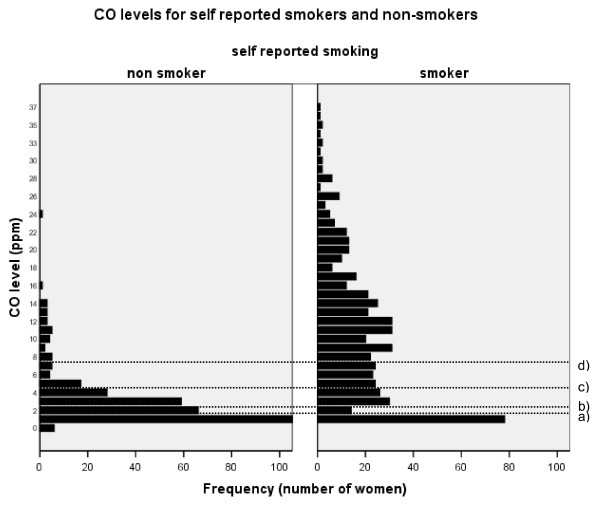
**Bar Chart of CO measurements for non-smokers and smokers**. The four lines indicate a) and b) 2 and 3 p.p.m.: the cut-off points with highest sensitivity and specificity c) 5 ppm: the new cut off for the Breathe programme and d) 8 ppm: the current cut-off point. It is possible to visualise the increase in number of subjects picked up by the test as the cut-off point is lowered. Note: The scale for the frequency of women has been truncated so as to remove the large peak of non-smokers at 1 p.p.m. (n = 1790). This allows the results for all women to be visualized more appropriately.

Of the 2661 women in the 'Breathe' dataset 2650 (99.6%) had self reported smoking data and 2548 (95.8%) had both self reported smoking data and CO validated measurements. Of these 2548 women, n = 2002 (78.6%) reported they were non-smokers and n = 546 (21.4%) were current smokers. 13.9% (n = 354) of women had a CO level of = 8 p.p.m. and were categorised by the current cut-off as validated smokers. The CO breath test results and self reported smoking status for all subjects are shown (Table 1, Figure 1).

**Table 1:** CO breath test result and self reported smoking status for all subjects

CO breath test	CO level	Non smokers (number)	Smokers (number)				Total number
			0–10	11–20	21–30	>30	

	0	6	0	0	0	0	6
	1	1790	70	6	0	0	1866
	2	66	11	4	0	0	81
	3	59	26	4	0	0	89
	4	28	23	2	1	0	54
	5	17	15	9	0	0	41
	6	4	20	2	1	0	27
	7	5	14	8	3	0	30
	≥8	27	177	114	30	6	354
	Total	**2002**					**2548**

Twenty-seven women with a CO of ≥ 8 p.p.m. described themselves as non-smokers (98.7% specificity); whereas 219 women with CO levels < 8 ppm reported they were smokers (59.4% sensitivity). Median CO level was 1 p.p.m. for non-smokers and 10 p.p.m. for smokers.

### Alternative Carbon Monoxide cut-off levels

**Figure 2:**
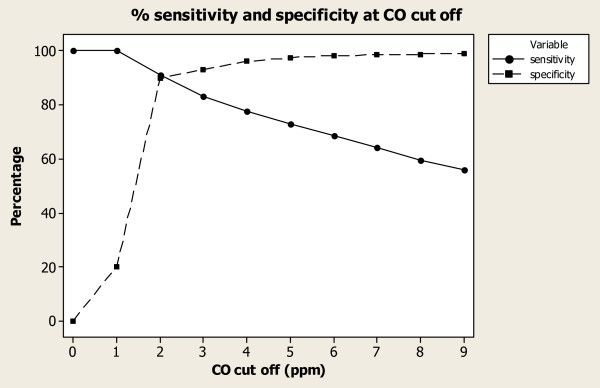
**Sensitivity and specificity curves**. CO cut-off levels from 1 to 9 p.p.m. were plotted. Definitions for sensitivity and specificity can be found in the Methods.

As the CO cut-off increased in value from 1 to 12 p.p.m., sensitivity decreased and specificity increased (Table 2). The highest average for combined sensitivity and specificity (88%) was observed at a CO cut-off level of 2 and 3 p.p.m. When plotted (Figure 2), the sensitivity and specificity curves intersect at a CO level between 2 and 3 p.p.m. This intersection indicates that the highest combined sensitivity and specificity was observed at a CO cut-off level less than 3 p.p.m.

**Table 2:** Sensitivity and specificity of various CO cut-off levels

**CO cut-off (p.p.m.)**	**Sensitivity**	**Specificty**	**(Sensitivity + specificity)/2**
**0**	1	0	0.5
**1**	1	0.2	0.6
**2**	0.857	0.897	**0.88**
**3**	0.832	0.930	**0.88**
**4**	0.777	0.960	0.87
**5**	0.729	0.974	0.85
**6**	0.685	0.982	0.83
**7**	0.643	0.984	0.81
**8**	0.594	0.987	0.79
**9**	0.559	0.989	0.77
**10**	0.502	0.990	0.75
**11**	0.465	0.992	0.73
**12**	0.408	0.995	0.70

At a CO cut-off level of 2 p.p.m, 1796 of the 2002 non-smokers had negative tests (specificity = 90 %), whereas 468 of 546 smokers had positive tests (sensitivity = 86%). This contrasts sharply with a CO cut-off level of 8 p.p.m. where, 1975 of 2002 non-smokers had negative tests (specificity = 99%), but only 327 of 546 smokers had positive tests (sensitivity = 60%).

### Relationship between cigarettes smoked per day and CO levels

**Figure 3:**
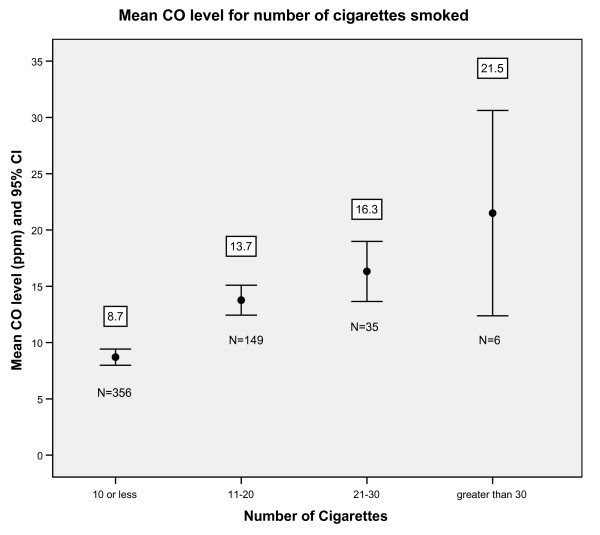
**Mean CO levels for each cigarette category with 95% confidence intervals for all reported smokers (N = 546)**. The mean CO value for all smokers in each category is shown above the bar. The minimum and maximum CO levels for those smoking <10, 11–20, 21–30 and 30+ cigarettes are, 1–34, 1–35, 4–37 and 13–36 p.p.m. respectively.

The mean CO levels increase with increasing number of reported cigarettes smoked (Figure 3). The maximum and minimum CO values illustrate the overlap for CO levels between the categories for numbers of cigarettes smoked.

## Discussion

The current paper examines the relationship between self-reported smoking status and validated smoking status using the Carbon Monoxide (CO) breath test. Results show that, with a sensitivity of 60%, the cut-off level of 8 ppm used for CO tests in the antenatal booking clinic is insufficiently sensitive. Of the 546 women who reported to be smokers 219 had CO values <8 ppm. Other than the possibility of data input error by midwives and nurses, there i, s no reason to presume women would say they smoke when they do not. It seems likely, therefore, that their smoking levels were too low to be detected and that the cut-off point for the CO test to improve detection of smokers requires adjusting downwards. This is especially important as light smokers are more likely to respond to smoking cessation intervention. It is important to recognise that breath CO has a short half-life of approximately 1–4 hours and thus it may not detect low levels of smoking [14]. In this study, the lower level of cigarette smoking in women who did not reach a cut-off of 8 ppm reflects this.

Evaluation of alternative cut-off levels pointed to a CO cut-off of 2 or 3 p.p.m. providing the highest accuracy for assessment of abstinence of smoking in this population. This cut-off was confirmed by several analyses. When the cut-off level was lowered, the sensitivity and specificity for self-reported smoking against CO, at 86% and 90% for 2 p.p.m. and 83% and 93% for 3 p.p.m., respectively, were high. This contrasts to the lower average of combined sensitivity and specificity observed at a cut-off of 8 ppm. Javors et al [17] recently suggested that a cut-off of 2 to 3 p.p.m. is more appropriate for detecting smokers within the general population. The current paper concurs with cut-off levels of 2 ppm and 3 ppm in the antenatal clinic setting to detect, with reasonable accuracy and discrimination, women who are smokers.

### Accuracy of self reported smoking

There is a caveat with regard to the use of self reported smoking to establish the CO cut-off. In Scotland the proportion of pregnant women smoking at booking is approximately 25% and in Glasgow this figure is even higher at 31.2% [19]. The proportion of self reported smokers in the current study population was 21% indicating that at least 10% of women in this population are potentially not telling the truth about their smoking status. This inaccuracy in reporting could explain why many reported non-smokers had CO levels beyond 2–3 p.p.m. and why 27 reported non-smokers had readings ≥ 8 p.p.m. There were some interesting comments made by women who reported as non-smokers but who had high CO levels:

• "..sitting in front of a smoker on the bus here" (24 ppm)

• "Gas fire needs fixing" (13 ppm)

• "Walked under the bridge on the way here – a lot of pollution" (9 ppm)

There are suggestions that changes in pregnancy may increase CO absorption from non-smoking sources, particularly later in pregnancy [20]. However, the contribution of environmental smoke exposure is multifactorial and difficult to assess: the reported cause is unlikely to be a sound measure of exposure. At such high levels one can almost be certain these women are denying their smoking status and that reporting exposure to environmental sources may have offered a "way out" for some women who did not want to admit to smoking. The Information Services Division for Scotland (ISD) states that the data on smoking behaviour are: "...based on self-reported information obtained from mothers at their ante-natal booking visit in the community or at hospital." The lower prevalence of smoking found in this study population may indicate that self reported smoking is not accurate in the antenatal clinic setting. Since the ISD use self reported smoking at the antenatal booking clinic to quantify the number of pregnant smokers, they are potentially under-estimating levels of smoking in the Scottish population. Thus, self reported smoking is an imperfect method of recording smoking status in this population.

### CO p.p.m. versus self reported intensity of smoking levels

There is considerable overlap in CO levels for all categories of number of cigarettes smoked. For example, a woman smoking 21–30 cigarettes could have the same reading, as low as 4 ppm, as a woman smoking 10 or fewer cigarettes. Time since last cigarette and CO testing and also underreporting of number of cigarettes smoked requires further study with direct measures such as cotinine to support findings in this study that the CO test is poor at distinguishing between different intensities of cigarette smoking. Since the CO means are noticeably different for the various smoking categories the CO test may be useful for showing only major changes in reduction of cigarette intake, and this could be used as a motivational tool.

It is recognised in current literature that light smokers (women smoking less than 10 cigarettes per day) are significantly more likely to quit during pregnancy [21]. It is therefore essential that any screening programme identifying smokers must pick up with accuracy, this population of pregnant smokers.

### Policy Issues

It has been documented in studies in New Zealand and the US that at maternity booking or at the end of pregnancy, over 20% of pregnant smokers falsely categorise themselves as non-smokers when asked by their midwife. This is the method used in the UK to identify pregnant smokers in order to refer them for specialist smoking cessation support. [22]

The under-reporting is likely to result from fear of disapproval rather than because these women don't want to stop smoking. There is a recognised policy in the UK to establish services for pregnant smokers. If the identification system (self-report at maternity booking) misses out 20% of pregnant smokers, by not recognising them, they are denied an important service. It is therefore important to identify their smoking habit in a less judgmental fashion: perhaps in the same way that all women in the UK are screened at maternity booking for syphilis by testing their blood samples. Our study shows that CO breath testing at the recognised cut-off does not accurately detect smokers, particularly low level pregnant smokers who are a target group. Another biochemical marker such as cotinine in blood or urine may be required to identify all pregnant smokers so they can benefit from specialist smoking cessation support and to assess the effectiveness of interventions. The role of clinicians is not necessarily to ensure the success of making all smokers quit, but rather to provide a reliable cessation service that is offered to all those who smoke. Increasingly, smoking cessation services are using the CO breath test for verification of non-smoking. The Breathe Tobacco Planning Interest Group state that the importance of the CO test is in its ability to demonstrate an immediate and potentially harmful consequence of smoking, and, consequently, to increase the number who take part in the programme and comply with advice to quit. By missing a large proportion of smokers, the test does not achieve this effectively. As a consequence of the current research the Planning Group have changed policy in antenatal clinics and reduced the CO cut off point to ≥ 5 ppm. This cut-off was seen as most appropriate when balanced with available resources and in future may be reduced further.

### Limitations

It was not possible to control for the issue of time since last cigarette and as a result it is possible that a woman who smokes prior to the clinic will have a very different CO reading to a woman who smokes after the clinic. It is also possible that women who self reported as non-smokers may have been light smokers, and may not have exceeded a CO level of 2 or 3 ppm; thereby reducing further the correlation between self report and biochemical measures. Lower CO levels observed during pregnancy do not necessarily reflect less smoke exposure, and cut-off levels used to classify non-smokers, passive smokers, and active smokers need to be established for pregnancy. It can be expected that the quality of measurement performed by midwives is variable. A further limitation was the use of participant self-report as the "gold standard" to determine smoking status. Cotinine or thiocyanate are possible alternative biomarkers of smoking for future study.

## Conclusion

At a cut-off of 3 ppm 113 (4.4%) more women would have been offered smoking cessation advice. The consequences of referring non-smokers depend on the number wrongly being referred. At 3 ppm a total of 113 of self-reported "non-smokers" will be contacted. Midwives in the service state that this is not a problem as most women will state this when contacted and no further contact will be made by the midwife. The impact on finances and resources is an issue. To accurately assess this it would be necessary to consider the number of women wrongly being referred versus the long term benefits of contacting a denier who then proceeds to use the service and quit. The costs of contacting all women, "a blanket referral", would most certainly outweigh the costs of carrying out a CO test and therefore an appropriate cut-off is important to maximise cost-benefit outcomes.

At 8 p.p.m., many smokers are missed and there may be gross underestimating of levels of smoking in a pregnant population. Results emphasise the need for further research to support a lower cut-off level for the breath CO test closer to 2 or 3 p.p.m. This may be more appropriate in the antenatal clinic setting, and is in line with recent recommendation in the non-pregnant population.

## Authors' contributions

ZCU carried out the research, and wrote the manuscript. PC participated in the supervision and coordination of the project. DS performed the statistical analysis. DT participated in the design and coordination of the study and helped to draft the manuscript. All authors read and approved the final manuscript.
